# Changes in Protein Glycosylation in Head and Neck Squamous Cell Carcinoma

**DOI:** 10.7150/jca.51604

**Published:** 2021-01-01

**Authors:** Chengcheng Liao, Jiaxing An, Zhangxue Tan, Fangping Xu, Jianguo Liu, Qian Wang

**Affiliations:** 1 Oral Disease Research Key Laboratory of Guizhou Tertiary Institution, School of Stomatology, Zunyi Medical University, Zunyi 563006, China.; 2 Department of Gastroenterology, Affiliated Hospital of Zunyi Medical University, Zunyi 563000, China.; 3 Microbial Resources and Drug Development Key Laboratory of Guizhou Tertiary Institution, Life Sciences Institute, Zunyi Medical University, Zunyi 563006, China.

**Keywords:** Glycosylation, HNSCC, EGFR, E-Cadherin, CD44, PD-1/PD-L1, B7-H3, Muc1

## Abstract

Glycosylation is an important posttranslational modification of proteins, and it has a profound influence on diverse life processes. An abnormal polysaccharide structure and mutation of the glycosylation pathway are closely correlated with human cancer progression. Glycoproteins such as EGFR, E-cadherin, CD44, PD-1/PD-L1, B7-H3 and Muc1 play important roles in the progression of head and neck squamous cell carcinoma (HNSCC), and their levels of glycosylation and changes in glycosyl structure are closely linked to HNSCC progression and malignant transformation. The regulation of protein glycosylation in HNSCC provides potential strategies to control cancer stem cell (CSC) subgroup expansion, epithelial-mesenchymal transition (EMT), tumor-related immunity escape and autophagy. Glycoproteins with altered glycosylation can be used as biomarkers for the early diagnosis, monitoring and prognostication of HNSCC. However, the glycobiology of cancer is still a new field that needs to be deeply studied, especially in HNSCC.

## Introduction

Head and neck squamous cell carcinoma (HNSCC) refers to squamous epithelial malignancies that originate in the oral cavity, nasopharynx, oropharynx, hypopharynx and larynx. The total incidence of HNSCC ranks sixth among neoplastic diseases, consisting of more than 90% of all head and neck tumors 1. Globally, there are approximately 600,000 new cases of HNSCC each year, most of which are locally advanced 1,2.] The 5-year survival rate of HNSCC patients is approximately 43%, and most patients cannot obtain an early diagnosis even after the tumor has metastasized to the lymph nodes in the neck 1. Therefore, it is important to explore effective molecular diagnostic indicators and design new treatment strategies for HNSCC patients with various tumor stages.

**Figure 1:**
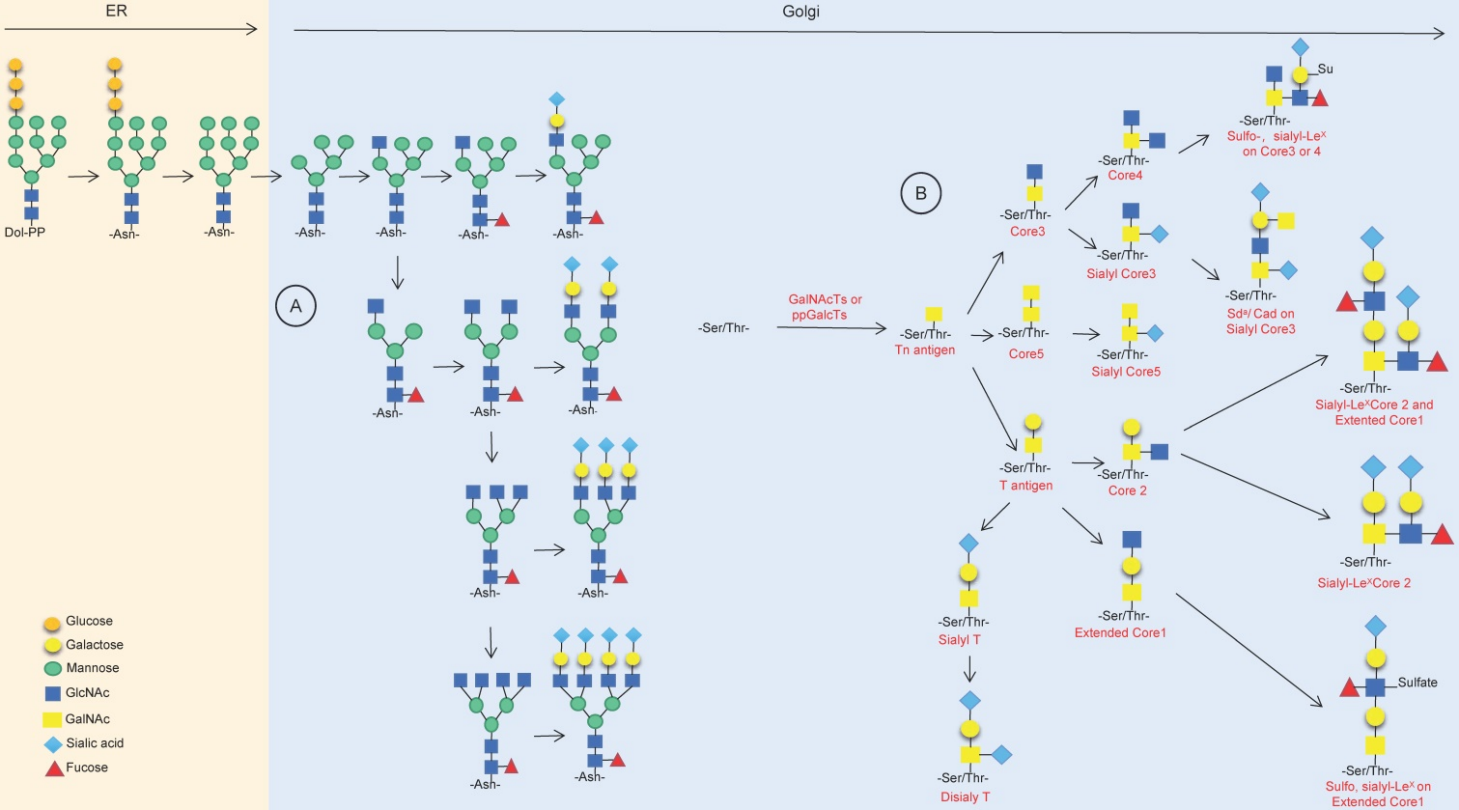
** Synthesis of N-linked glycans and O-linked glycans.** (A) Synthesis of mainly asparagine streptan (N-glycan) in the endoplasmic reticulum and Golgi apparatus. (B) Synthesis of O-linked glycans and common products in the Golgi.

Protein glycosylation occurs during the process of peptide chain synthesis, and the sugar chain is linked to a specific glycosylation site on the peptide chain via enzyme catalysis. There are 9 kinds of glycans in mammalian cells, including glucose, N-acetylglucosamine, galactose, N-acetylgalactosamine, mannose, fucose, gluconic acid, xylose and sialic acid. The most common advanced glycation end products (AGEs) are N-glycosylation and O-glycosylation 5,6. ]More than 2,000 proteins in human cells harbor an amino acid motif suitable for N-glycosylation, and these include membrane-bound proteins and secreted proteins but do not include cytoplasmic or nuclear proteins 7. N-glycosylation occurs during the translation of target proteins by appending glycan structures to the amino group of asparagine (Asn); it starts in the endoplasmic reticulum (ER) and ends in the Golgi body, forming di-, tri- or tetraantennary glycans and then decorating them with various modifications, including fucosylation, salivation, galactose addition and GlcNAc, finally forming a series of highly complex and nonuniform structures of N-glycosylation (**Fig. 1A**) 7-10. O-glycosylation happens in the Golgi apparatus; with the catalyst of polypeptide-GalNAc-transferases (pp-GalNAcTs, GALNTs), a single N-acetylgalactosamine (GalNAc) residue is transferred to serine (Ser) and/or threonine (Thr) residues of specific proteins, forming the Tn antigen, which finally forms five core structures named core 1 to core 5 (**Fig. 1B**) 7,11-13.

Glycosylation may represent a hallmark of cancer 14 and causes the secretion of tumor-associated polysaccharides or glycoproteins, promoting their release into the bloodstream as a tumor-related marker 15. The glycosylation process elevates the complexity of protein function regulation and has therapeutic significance for many diseases, including cancer, in which it regulates the proliferation, invasion and angiogenesis of tumors 16,17. ]Alterations in glycosylation directly affect tumor growth and survival and facilitate tumor-induced immunomodulation and eventual metastasis 18. Accurate diagnosis and treatment based on tumor glycosylation is a hot topic in biomedical research. Understanding HNSCC-related glycosylation alterations is critical for developing therapeutic interventions that aim to recover the normal glycosylation patterns of cancer cells. In this review, we focused on changes in protein glycosylation and its effects on HNSCC and discussed aberrant glycosylation levels and their potential use as diagnostic markers of HNSCC. According to the latest studies of protein glycosylation, this review introduces recent advances in EGFR, E-cadherin, CD44, PD-1/PD-L1, B7-H3 and Muc1.

## The effect of EGFR glycosylation on the biological behavior of HNSCC

Epidermal growth factor receptor (EGFR/HER1/ErbB1) belongs to the tyrosine kinase receptor ErbB family 19. It is overexpressed in most epithelial-derived malignancies, such as HNSCC 20. EGFR is involved in many crucial signaling pathways, such as RAS/RAF/MEK/MAPK/ERK, PI3K/Akt, and JAK/STAT. These signaling cascades widely influence tumor growth, metastasis, poor prognosis, resistance to chemotherapy and radiation in HNSCC 21,22. ]EGFR is widely expressed in HNSCC with few genetic mutations, making it an attractive therapeutic target for HNSCC patients who have a poor prognosis and are prone to recurrence and metastasis 23. Understanding the effect of EGFR glycosylation on the interaction between EGFR and its related ligands will help us to elucidate the activation mechanisms of EGFR and facilitate the computational design of efficient inhibitors.

### EGFR N-glycosylation correlation with malignant progression of HNSCC

N-glycosylation influences both the structure and stability of EGFR. The N-glycosylation of the glycosyl group next to the EGF binding site in EGFR could cause its noncovalent interactions with the EGFR extracellular domain, stabilizing the EGF binding site, leading to a stronger interaction between EGF and EGFR; N-glycosylation also helps maintain the binding of the EGFR dimer and plays a special role in the binding of antibodies and the extracellular domain of EGFR 24.

The N-glycosylation inhibitor 2-deoxyglucose (2DG) could change the N-glycosylation state of EGFR in HNSCC, induce the expression of the endoplasmic reticulum stress (ERS) markers CHOP and BiP, and finally affect the activity of EGFR; therefore, 2DG can enhance the antitumor effects of cisplatin and radiotherapy and overcome erlotinib resistance 25. In addition, the disruption of glycolysis by 2DG is also a way to exert a tumor-suppressive effect 26,27. Tunicamycin (TM), which is another N-glycosylation inhibitor, transfers N-acetylglucosaminyl-1-phosphate to phosphorylated dolichol, inhibiting N-linked glycosylation by blocking GlcNAc phosphotransferase (GPT) 28. Wang et al. 29 found that after incubation with TM, ERS was increased and the EGFR signaling pathway was suppressed by inhibition of EGFR N-glycosylation.

**Figure 2:**
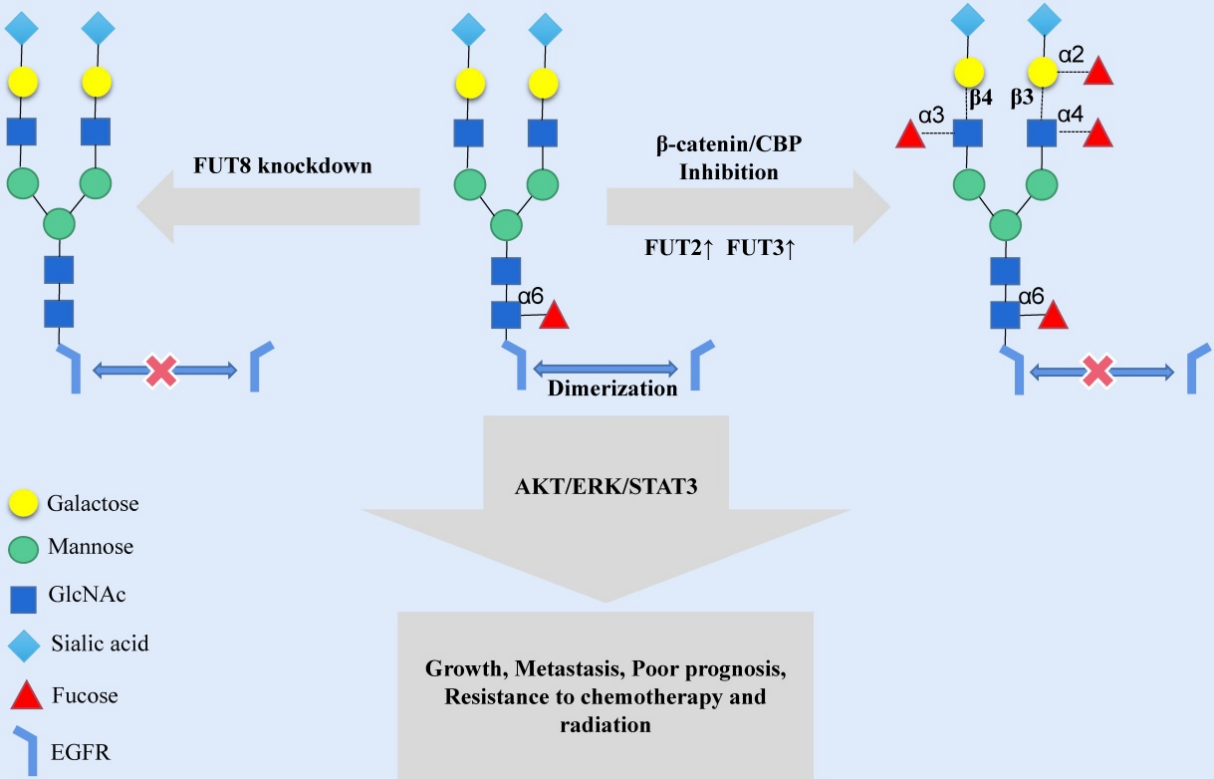
Increasing the expression of FUT2 and FUT3 or decreasing the expression of FUT8 in the Golgi can destroy the extracellular N-glycan structure of EGFR protein and affect the transmission of EGFR signal cascade.

Modifying the terminus of N-glycosylation is another way to change the N-glycosylation-mediated activity of EGFR. Human tumor-specific N-glycan modifications include fucosylation, salivary acidification and lactose addition 7. The fucosylation of N-glycans is mainly catalyzed by fucosyltransferase (FUT); the FUT family is a group of fucosylation synthetases, including FUT1 to FUT11, which catalyze the transfer of fucose from GDP-fucose to oligosaccharides on the substrate, sugars of glycoproteins chain or glycolipid 30. In human malignant oral squamous cell carcinoma (OSCC) cells, the fucosylation of N-glycan antennae is lacking in EGFR, while indolent cells have high levels of fucosylation at the N420 and N579 sites of EGFR. ICG-001 and E7386, inhibitors of β-catenin/CBP signaling, may cause an increase in the transcription expression of FUT2 and FUT3 and then increase the fucosylation level of N-glycan antennae in EGFR 31 (**Fig. 2**). The structural modification of EGFR N-glycan might trigger a change in the cellular localization and signal transduction of EGFR. FUT1 is a key enzyme for Lewis^Y^ (Le^y^) synthesis 32, and the Le^y^ precursor is related to the motility of oral mucosa cells 33. The increased expression of Ley was significantly associated with poor prognosis, and Le^y^ of EGFR could stabilize the expression EGFR and downstream signals and promote the migration of OSCC cells 34. FUT4 is involved in the synthesis of Lewis^X^ (Le^X^) in HNSCC, and Le^X^ is related to the drug resistance response and the prognosis of HNSCC patients 35. FUT8 is a typical N-glycan branching enzyme and plays a critical role in regulating the signal transduction of cell surface receptors 36. Although FUT8 does not affect the expression of EGFR on the cell membrane, the FUT8-mediated core fucosylation of N-glycans is necessary for EGF to bind its receptor, and the lack of FUT8 could influence the activity of EGFR (**Fig. 2**). However, it should be noted that the inhibition of FUT8 may considerably impact normal human cells, thus limiting the application of FUT8 inhibitors in tumor therapy 37.

Abnormal sialylation of EGFR is associated with the malignancy, metastasis and aggressiveness of tumors 38. Sialylation could suppress EGFR dimerization, autophosphorylation, and EGF-induced lung cancer cell invasion 39. When compared with the primary tumor of HNSCC, the sialic acid content in metastatic tumors is lower 40. Sialidase regulates cellular sialic acid by removing the a-glycosidically linked sialic acid residues of glycoproteins and glycolipids and is involved in the development of cancer. The plasma membrane-associated sialidase NEU3 plays a unique role in transducing EGFR transmembrane signals by regulating the hydrolysis of gangliosides 41. NEU3 could enhance the phosphorylation of EGFR and promote the migration and invasion of HNSCC cells. The expression of NEU3 is significantly upregulated in HNSCC tissues compared with in normal epithelial tissues, and its increased mRNA is positively correlated with lymph node metastasis 42. According to the above results, NEU3 might possess the ability to increase the malignancy of HNSCC cells by inhibiting sialylation on EGFR.

To date, cetuximab is the only FDA-approved EGFR-targeted therapy drug for the treatment of HNSCC 43. However, the treatment efficacy of cetuximab is low, with an objective response rate of 13% as a monotherapy and 36% in combination with chemotherapy 44,45.] Friederike et al. 46 reported that after chemotherapy or radiotherapy combined with cetuximab, HNSCC patients with higher EGFR-K521 (k-allele) expression levels had a significantly shorter progression-free survival period compared with those without expression of EGFR-K521. Cetuximab cannot inhibit the downstream cascade of the EGFR signaling pathway in HNSCC cells with high k-allele expression. EGFR-K521 N-glycan is sialic acid deficient compared to EGFR protein, and EGFR-K521 protein N-glycan sialic acid deficiency may be a structural basis for reducing the effectiveness of cetuximab. This study was important for exploring the mechanisms of cetuximab resistance and suggested that N-glycan sialic acid deficiency EGFR-K521 can be used as a biomarker to predict the prognosis of HNSCC patients.

### Aberrant O-glycosylation interferes with EGFR vitality

GalNAc-type O-glycosylation is initiated by the transfer of N-acetylgalactosamine (GalNAc) to a serine or threonine residue and forms the Thomsen-nouvelle (Tn) antigen; this process is catalyzed by the GalNAc transferase family (GALNTs) 47,50. GALNT2 is overexpressed in cancer cells at the invasive front of OSCC and enhances the invasiveness of OSCC cells by modifying EGFR O-glycans 48. C1GALT1 (Thomsen-Friedenreich antigen) is the only enzyme that catalyzes the transfer of UDP-galactose to the Tn antigen and forms the core 1 structure 49; moreover, C1GALT1 is the precursor of various GalNAcl-type O-glycans that are extensively distributed on secreted glycoproteins and the cell surface. The knockdown of C1GALT1 could inhibit the O-glycan extension of EGFR, reduce the binding affinity of EGF-EGFR, and thereby inhibit the signal transduction of EGFR. Itraconazole is a C1GALT1 inhibitor and may be therapeutic for HNSCC 50. Although the O-glycosylation of EGFR seems to be less important than N-glycosylation, it is necessary to know that the change in EGFR activity is caused by aberrant O-glycosylation in HNSCC.

## The influence of aberrant glycosylation on cancer stem cells of HNSCC

Cancer stem cells (CSCs) are a small subgroup of tumor cells with the ability to self-renew and differentiate. HNSCC CSCs are highly tumorigenic and participate in tumor differentiation, treatment resistance, relapse and metastasis 51. In CSCs, the glycosylation of specific markers affects diverse fundamental processes of cells, such as adhesion, survival, invasion, metastasis, pluripotency, stemness, drug resistance and apoptosis 52. Therapy targeting glycosyl groups on CSCs combined with surgical treatment, radiotherapy and chemotherapy will help improve the prognosis of patients.

### The influence of glycosylated CD44 on the stemness of HNSCC CSCs

CD44 is a biomarker of CSCs and regulates cancer stemness in solid tumors 53. CD44 members contain CD44s (standard CD44) and CD44 v1-v10, and they both have unique cell adhesion properties that could cause the interaction between two different cell types or one cell type and its surrounding matrix, sequentially accelerating the aggregation and migration of tumor cells 54. CD44 can be glycosylated through N-glycans and O-glycans, and the molecular weight of the CD44 subtype depends on its glycosylation level 50,55.] The functional diversity of CD44 is derived from its various glycosylation patterns in the extracellular matrix and the multiple subtypes of protein produced by alternative splicing, which also contributes to the metastasis of human tumors 56,57.] The expression of CD44v4 leads to the activation of ERK1/2 and resistance to cisplatin. CD44v6 is mainly related to the activation of PI3K/Akt/GSK3β and the invasion and migration of HNSCC; CD44s and CD44v3 participate in the growth and migration of HNSCC 58,59 but the differences in CD44s and CD44 V1-V10 glycosylation structures and their corresponding functions have not been fully elucidated. TM can lessen the expression of CD44 in HNSCC by inhibiting N-glycosylation 30. In addition, when a glycosylated, conformation-dependent CD44 epitope is targeted by monoclonal antibody RO5429083, it can increase the number of natural killer (NK) cells, reduce the EGFR signaling of CSCs and increase the targeting efficiency of RO5429083 towards CSC populations 60.

Hyaluronic acid (HA) is one of the main components of the extracellular matrix. HA accumulation is closely related to the poor prognosis of patients with advanced cancer and is also conducive to tumor angiogenesis, invasion and metastasis 61. It has been proven that HA can stimulate the self-renewal, clonal formation and differentiation of CSCs 62. The extracellular N-terminal hyaluronic acid binding domain (HABD) of CD44 could prompt its bond to HA. The terminal sialic acid on CD44 N-glycan can form a charged paired hydrogen with the basic amino acid of HABD, interrupting the bond of HA to glycosylated CD44 HABD 63. In addition, the integrity of potential N-linked glycosylation sites of CD44 is critical for hyaluronic binding 64. The c-Jun signal induced by HA/CD44 leads to the production of survival protein (cIAP-1/cIAP-2) and chemotherapy resistance in HPV16^+^ HNSCC cells, thus promoting the development of HNSCC 65.

Except for CD44, more than 30 CSC markers can be used to identify different cancer and tissue types of CSCs, and most of them are cell surface glycoproteins 66. Although further investigations are still needed, some of these markers, in addition to CD44, may be affected by glycosylation changes. For example, when comparing the mass spectra and lectin binding in CD133^+^ and CD133^-^ cells, there was a greater than 10% difference in the N-glycan structures 67. When CSCs of colon cancer differentiate into tumor cells, the glycosylation of CD133 may change and lead to different folding patterns of CD133, thus covering different specific epitopes 68.

### The influence of altered fucosylation on the stemness of HNSCC CSCs

It has been proven that fucosylation is closely associated with pancreatic CSCs 69, and the expression of FUT9 in colorectal cancer cell lines is positively correlated with the phenotypic characteristics of CSCs 70. In addition, the miR-29b/Sp1/FUT4 axis can promote the malignant behaviors of leukemia stem cells by regulating fucosylated CD44 via the Wnt/β-catenin pathway 71. These results suggest that adjusting the level of fucosylation in tumor cells may affect the stemness of CSCs.

Sialyl Lewis-X (SLe^X^) is closely related to CSCs in HNSCC, while FUT3, FUT5 and FUT6 participate in the synthesis of SLeX 72. In OSCC CSCs, the expression of fucosyltransferases FUT3 and FUT6 on the spherical cell membrane is higher when compared with that in adherent cells, and the increased expression of SLe^X^ might lead to greater aggression, tumorigenicity, tumorigenicity, chemoresistance and lower radiosensitivity of CSCs 73. Moreover, the content of secreted SLeX is very high in OSCC and increases with the progression of disease. The SLeX positive rate in OSCC cells cultured in suspension is 95-100%, while that in adherent cells is 10-40% 74.

## Aberrant N-glycosylation status affects E-cadherin-mediated EMT

Epithelial cadherin (E-cadherin or cadherin 1) belongs to the cadherin family, and the reduction in E-cadherin expression is involved in the progression and metastasis tumors 75. E-cadherin is highly expressed in the normal squamous epithelial cells of the oral cavity and oropharynx and inhibits the disengagement-dependent growth and EMT transformation of HNSCC cells 76. The absence of E-cadherin might result in the dissociation of invasive tumor cells at the edge of tongue carcinomas and regional metastasis of lymph nodes 77. In normal epithelial cells, the inhibitory effect of E-cadherin against tumorigenesis is maintained by sequestering the binding of β-catenin to lymphoid enhancer factor (LEF)/T cell factor (TCF); this process could impede the transcription of genes involved in the proliferation-associated Wnt signaling pathway 78. The E-cadherin/*β-catenin* complex helps maintain the integrity of epithelial cells, disrupts the Wnt signaling pathway, and participates in a variety of human malignancies and fibrosis disorders caused by EMT 79.

The posttranslational modifications of E-cadherin include phosphorylation modification, O-glycan modification and N-glycan modification. β1,6-GlcNAc branched N-glycans are of great importance to the regulation of E-cadherin-mediated adhesion and signal transduction 80. The N-glycosylation of E-cadherin could influence the progression of tumors and transformation towards a malignant phenotype 81. N-acetylglucosamine transferase III (GnT-III), N-acetylglucosamine transferase V (GnT-V) and FUT8 are related to the reconstruction of E-cadherin N-glycan 82. Aberrant N-glycosylation at the Asn-554 83,84 Asn-566 84 and Asn-633 85 sites of E-cadherin could strengthen its critical function in cancer.

**Figure 3:**
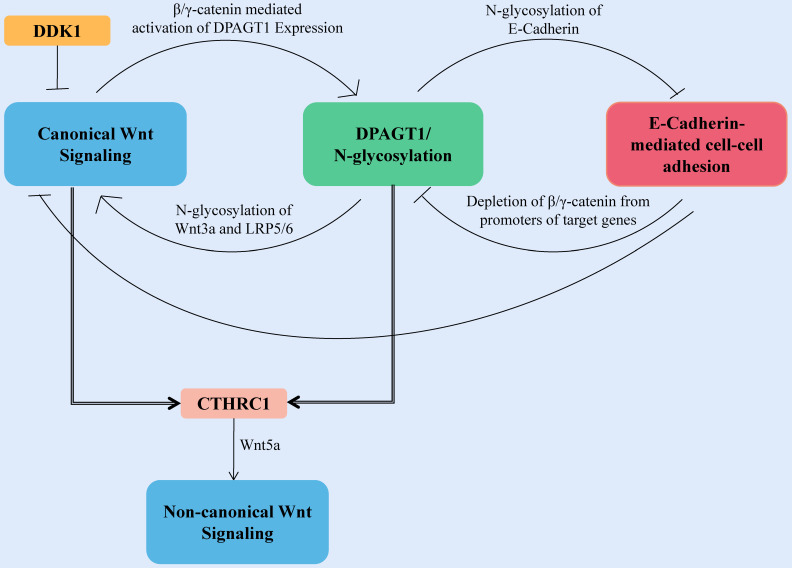
** Canonical Wnt signaling activates DPAGT1 expression and protein N-glycosylation, leading to extensive N-glycosylation of E-cadherin.** In HNSCC, the positive feedback loop between Wnt signaling and DPAGT1 is amplified and partially inhibited by wnt pathway inhibitor DDK1. Furthermore, extensive N-glycosylation of E-cadherin prevents it from depleting nuclear β/γ-catenins allowing the positive feedback between Wnt and DPAGT1 to operate without controls. CTHRC1 is upregulated by DPAGT1 and canonical Wnt signaling, affecting the noncanonical Wnt pathway.

The first committed step of protein N-glycosylation is catalyzed by the dolichyl-phosphate N-acetylglucosamine-phosphotransferase DPAGT1 86. DPAGT1 is a key node that regulates the loss of E-cadherin and the activation of the Wnt pathway induced by aberrant N-glycosylation-related networks (**Fig. 3**). DPAGT1 and Wnt/β-catenin control the N-glycosylation status of E-cadherin through positive and negative feedback mechanisms, reducing the localization of E-cadherin on the cytomembrane of HNSCC (**Fig. 3**) 87-90. The Wnt signal intensity is regulated by the N-glycosylation degree of Wnt3a and low-density lipoprotein-related receptors 5 and 6 (LRP5/6) because Wnt3a and LRP5/6 can be secreted and expressed effectively on the cell membrane only under proper N-glycosylation (**Fig. 3**) 91.

## Glycosylation-related immune checkpoints and HNSCC immune escape

Aberrant glycan structures and mutations of the glycosylation pathway are associated with the immune escape ability of tumor cells 92. Specific glycan signatures on tumor cells can be considered a novel type of immune checkpoink 93. In parallel, the glycosylation of tumor proteins produces neoantigens that masquerade as normal parts of the body to evade immune cells 93,94. PD-1, CTLA-4, TIM-3, IDO and other inhibitory immune checkpoints have been proven to participate in the construction of the HNSCC immunosuppressive microenvironment 95. Many immune checkpoints, such as PD-1 96, B7-H3 97 and TIM-3 96 are glycoproteins with varying degrees of glycosylation.

### N-glycosylated PD-1/PD-L1 and HNSCC immune escape

The programmed death 1 (PD-1)/programmed death-ligand 1 (PD-L1) axis could suppress antitumor immunity 98. PD-1 interacts with PD-L1 to inhibit the proliferation of T cells and the production of cytokines 99. PD-L1 combined with CD80 impedes the activation of T cells 100. PD-L1 protein stability, translocation and protein-protein interactions can be altered by glycosylation, phosphorylation, ubiquitination, sumoylation and acetylation 101. Current research indicates that N-glycosylation and ubiquitination are the major posttranslational modifications involved in the immunosuppressive activity of PD-L1 102.

Let-7a/b can inhibit PD-L1 glycosylation and promote PD-L1 degradation in HNSCC, and the process is achieved via the β-catenin/STT3 pathway 103. EMT can induce the N-glycosyltransferase STT3 through β-catenin transcription, stabilize the N-glycosylation of PD-L1 and increase its expression, finally helping CSCs escape from the immune system 104. Deglycosylation significantly improves the binding affinity and signal intensity of anti-PD-L1 antibodies to PD-L1, making quantitative clinical outcome predictions based on PD-L1 more accurate 105.

**Figure 4:**
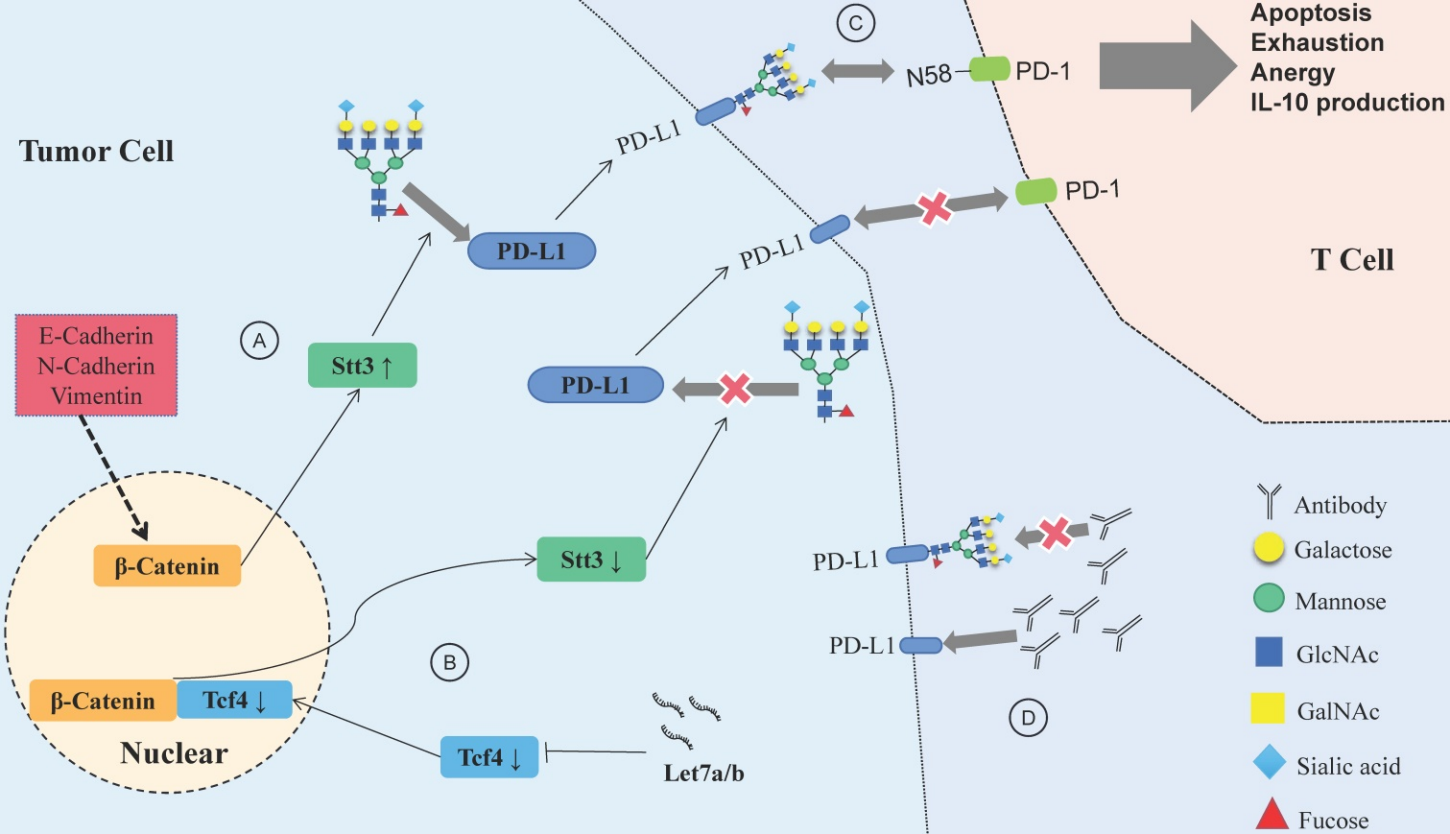
** Relationship between glycosylation changes and immune escape of HNSCC.** (A) E-cadherin, N-cadherin, and vimentin expression in HNSCC affects PD-L1 N-glycosylation, which is stabilized through the β-catenin/STT3 signaling axis. (B) The Let-7 family of miRNAs inhibits the expression of TCF-4, suppresses β-catenin/STT3-mediated PD-L1 glycosylation, reduces PD-L1 stability, promotes the ubiquitination and degradation of PD-L1, and improves the ability of T cells to recognize HNSCC cells. (C) Glycosylation of PD-1 mediates its interaction with glycosylated PD-L1, and the PD-1/PD-L1 axis has a central role in suppression of antitumor immunity. (D) Deglycosylation significantly improves the binding affinity and signal intensity of anti-PD-L1 antibodies to PD-L1.

N-glycosylation can stabilize the protein structure of PD-1, thus compromising the antitumor immune responses, while the inhibition of Fut8 can reduce the expression of PD-1 on the cell surface and enhance the activation of T cells, leading to more efficient cancer destruction 106. A recent study showed that PD-1 is extensively N-glycosylated in T cells; glycosylation of PD-1, especially at site N58, is the key to mediating its interaction with PD-L1 107. Therefore, inhibiting the glycosylation of PD-1/PD-L1 would help to suppress the immune escape of HNSCC and improve the efficacy of antibodies (**Fig. 4**). Designing antibodies against the glycosylation sites of PD-1/PD-L1 provides a potential way to confront PD-1/PD-L1-related immune escape.

### N-glycosylated B7-H3 and HNSCC immune escape

B7 homolog 3 (B7-H3), an immune checkpoint protein of the B7 family, is an important regulator of the adaptive immune response; B7-H3 is mainly expressed on the surface of tumor cells, antigen-presenting cells, NK cells, and tumor endothelial cells. B7-H3 has a common inhibitory effect on T cells and helps tumor cells escape from the immune system; it also participates in cell proliferation, migration, invasion, angiogenesis, metastasis and anticancer drug resistance 108,109.]

B7-H3 is overexpressed in OSCC, promoting aerobic glycolysis in OSCC via the PI3K/Akt/mTOR pathway 110. Compared with those of normal oral mucosal epithelial cells, the glycans of B7-H3 in OSCC contain terminal α-galactoses and more diverse N-glycan structures with higher fucosylation; with the action of B7-H3, OSCC cells could develop more effective interactions with DC-SIGN[DC-specific intercellular adhesion molecule-3 (ICAM-3)-grabbing nonintegrin] and Langerin on immune cells 111. These results suggest that glycosylation changes of B7-H3 affect the occurrence and development of OSCC and change the immune microenvironment of OSCC tumors.

## O-GlcNAc mediates HNSCC autophagy

The function of autophagy-related proteins, in particular their interaction with macroautophagic regulators, is modulated by phosphorylation, glycosylation, ubiquitination, acetylation, lipidation and proteolysis 112. Glycan signaling in the extracellular matrix affects the location, activity and/or expression of key autophagy regulators, such as AMPK and mTORC1. Within the intracellular space, the components of the autophagosome membrane include ganglion glycosides, a subset of proteins composing the autophagic machinery are regulated by glycosylation, and exposure of oligosaccharides to the cytoplasm can also trigger autophagy 113. The intracellular O-linked β-N-acetylglucosamine (O-GlcNAc) modification can regulate cell autophagy, as well as transcription, translation, protein degradation and signal transduction 114. It has been reported that O-GlcNAc regulates autophagy by modifying autophagy core protein Beclin 1 115, the forkhead family of transcription factors 116, SNARE protein SNAP-29 117, and autophagy modulators such as Bcl-2 115, AMPK 118. In HNSCC, the influence of various hypoxia factors and the expression pattern of HIF-1α leads to an increasing number of CSC subgroups, driving tumor growth, invasion and therapy resistance 119. In addition, HIF-1α inhibitors can suppress the autophagy of OSCC by decreasing the expression of O-GlcNAc and O-GlcNAc transferase (OGT) and increasing the expression of O-GlcNAcase (OGA) 120. However, in another study, the levels of O-GlcNAcylation did not increase significantly in OSCC tissues, which was also not connected with the histological grading of OSCC 121.

## Aberrant MUC1 glycosylation in HNSCC

MUC1 is a heterodimeric glycoprotein consisting of a highly glycosylated extracellular part and a small cytoplasmic tail 122. Due to the higher expression of MUC1 in OSCC than in normal mucosal tissues, it is considered a reliable biomarker for the diagnosis of OSCC. Silencing the *MUC1* gene could induce apoptosis and inhibit the proliferation, invasion, migration and EMT of OSCC cells 123,124. T]his membrane-localized glycoprotein is overexpressed and aberrantly glycosylated in most epithelial cancers 125, and the aberrant glycosylation of MUC1 might result in the shortening of the sugar chain, causing the exposure of hidden antigens; the hidden antigens usually have peptidic and carbohydrate properties, making the MUC1 epitope tumor-specific 126. In addition, extensive O-glycosylation of MUC1 contributes to cell resistance to anoikis, increasing cell adhesion and modulating the tumor immunological microenvironment through engagement of the lectin Siglec-9 127,128. Glycosylated MUC1 also has a positive sense and could stabilize an extended bioactive conformation of the peptide recognized by the antibody 129.

The biochemical functions and characteristics of the MUC1 protein have been identified. Due to the highly variable structure of MUC1, clarifying the specific effect of various MUC1 subtypes on tumor cells may have greater clinical significance 125. N-glycosylated MUC1-C contributes to the upregulation of galectin-3 expression and interaction of galectin-3 and MUC1 could promote the dimerization and activation of EGFR in human epithelial cancer cells 130,131.] In addition, phosphorylation of the MUC1-C cytoplasmic domain could regulate the function of MUC1-C in the Wnt/β-catenin pathway 132.

## Potential clinical applications of aberrant glycosylation as an HNSCC biomarker

Based on the abnormal glycosylated type of HNSCC, we can design corresponding treatment protocols and identify potential diagnostic biomarkers. Specific N-glycopeptide could be used as a serum biomarker to identify the clinical status of HNSCC patients. For the N-glycopeptides of IgG1, IgG4, HPT and TRFE, their abundances are significantly different between patients and controls, making them ideal candidates for future diagnostic modalities of OSCC 133. In another study, the relative abundances of fucosylated, triantennary and tetraantennary glycans were significantly increased in OSCC patient serum compared with normal human serum, and OSCC patients showed significantly elevated levels of two IgM antibodies and decreased levels of nine IgG antibodies (**Table 1**) 134.

Carcinoembryonic antigen-related cell adhesion molecule 6 (CEACAM6), a glycophosphoinositol-anchored protein, is a heavily glycosylated tumor antigen. N-glycosylated CEACAM6 protein is a tumor marker for early recurrence in OSCC patients. In addition, the complex N-glycosylation of CEACAM6 is essential for EGFR-mediated OSCC cell invasion and transfer 135. As mentioned above, aberrantly glycosylated proteins in serum and N-glycosylated CEACAM6 could be used as potential biomarkers for the diagnosis of OSCC. These biomarkers with abnormal glycosylation are summarized in **Table 1.**

## Conclusion and prospects

The posttranslational modification of proteins is a major proteomics challenge in the post-gene era, and glycosylation lies in the heart of the problem. We found that the glycosylation changes of EGFR, E-cadherin, CD44 and PD-1/PD-L1 and the other glycoproteins have profound impacts on EMT, stemness, immune escape and other key metabolic steps of HNSCC. However, these studies have not fully revealed the detailed effects of protein glycosylation changes on HNSCC. For example, it is acknowledged that there are various O-glycan-decorated Notch receptors distributed in the extracellular domain epidermal growth factor-like (EGF) repeats 136, and many human congenital diseases are caused by O-glycosylation defects on Notch receptors; however, the relationship between the O-glycosylation defect of the Notch receptor and HNSCC has not been reported. Due to the complex mechanisms and structures of glycosylation and the large number of glycoproteins, the understanding of glycosylation in HNSCC still has a long way to go.

The glycobiology of HNSCC is an important but understudied field. Initially, monoclonal antibodies were used to identify tumor-related glycosyl changes; currently, more advanced technologies, such as ultrahigh-performance liquid chromatography (UPLC), mass spectrometry (MS), glycan microarrays, lectin histochemistry and agglutination cell count, are used to identify tumor polysaccharides 137,138. These emerging research methods integrate glycomics, proteomics, genomics, lipidomics and metabolomics, making the study of glycobiological systems possible 139. In the past, the basic methods of analysis, verification and construction of glycoproteins developed slowly. The emergence of electron cryomicroscopy provides an efficient tool to study the key structures of glycobiology 140. However, these emerging methods have rarely appeared in existing HNSCC-related research.

The glycopeptide expression levels in the serum of HNSCC patients and changes in the protein glycosyl structure can be used as potential diagnostic indicators. IgG, HPT, and TRFE are abundant in serum, making them easier to identify. It is an expectation for us to diagnose HNSCC by assessing aberrant protein glycosylation, and the achievement of the goal should depend on the establishment and improvement of the glycosylation database. Moreover, it is feasible to design corresponding antibodies according to the target glycosyl sites of glycoproteins to treat HNSCC; in addition, regulating glycosyltransferase expression in HNSCC is also an important pathway for tumor suppression.

## Figures and Tables

**Table 1:** Aberrant glycosylation markers with clinical application value in OSCC

Antibody class	Glycan name	*P*-value	Application	Compared with normal level	Reference
IgM	SSEA3	< 0.01	Potential candidate OSCC biomarkers	↑	134
	CD2	0.005		↑	
IgG	GHC	0.01		↓	
	LeY	0.01		↓	
	SiaLeX	< 0.001		↓	
	αNeuAc-OCH2C6H4-p-NHCOOCH2	0.04		↓	
	NeuAcα2-8NeuAcα (NeuAcα2-8)2	0.01		↓	
	NeuAcα2-8NeuAcα2-8NeuAc (NeuAcα2-8)3	0.001		↓	
	Neu5Acα2-3Galβ1-4(Fucα1-3)(6-HSO3)GlcNAcβ (6GlcNAc-HSO3-SiaLeX)	< 0.01		↓	
	(NeuAcα2-6Gal1-4GlcNAc1-2Man)2α1-3,6Manα1-4GlcNAcβ1-4GlcNAc (α2-6 sialylated diantennary N-glycans)	0.001		↓	
	GD2	0.02		↓	
IgG1	N-glycan	Unknown but < 0.05	Diagnosis of OSCC as early as stage I	↑	133
IgG4				↑	
HPT				↓	
TRFE				↓	
EGFR-K521	Sialyl glycan	0.049	Prediction of the prognosis of patients with HNSCC	↑	46
CEACAM6	N-glycan	0.0276	Diagnosis of early recurrence in OSCC patients	↑	135

Note: The *P*-value is the comparison between normal samples and OSCC patient samples. ↑, Increased compared to normal levels; ↓, decreased compared to normal levels; -, unchanged from normal levels.
